# Free *N*‐heterocyclic carbenes from Brønsted acidic ionic liquids: Direct detection by electrospray ionization mass spectrometry

**DOI:** 10.1002/rcm.9338

**Published:** 2022-07-07

**Authors:** Chiara Salvitti, Federico Pepi, Marta Managò, Martina Bortolami, Cinzia Michenzi, Isabella Chiarotto, Anna Troiani, Giulia de Petris

**Affiliations:** ^1^ Dipartimento di Chimica e Tecnologie del Farmaco Sapienza Università di Roma Rome Italy; ^2^ Dipartimento di Scienze di Base e Applicate per l'Ingegneria Sapienza Università di Roma Rome Italy

## Abstract

**Rationale:**

The occurrence of *N*‐heterocyclic carbenes in imidazolium‐based ionic liquids has long been discussed, but no spectroscopic evidence has been reported yet due to their transient nature. The insertion of an ionizable acid group into the cation scaffold of an ionic liquid which acts as a charge tag allows for the direct detection of free carbenes by mass spectrometry.

**Methods:**

Three different Brønsted acidic ionic liquids were synthesized: 1‐methyl‐3‐carboxymethylimidazolium chloride (MAICl), 1‐methyl‐3‐carboxymethylimidazolium acetate (MAIAc) and the corresponding 2‐(3‐methyl‐1*H*‐imidazol‐3‐ium‐1‐yl)acetate zwitterion (MAI − H). The speciation of these compounds was then analysed by electrospray ionization ion‐trap mass spectrometry in the negative ion mode.

**Results:**

The C2‐H deprotonation of the imidazolium cation leading to the formation of the corresponding carbene is highly affected by the basic properties of the counter‐anion. In the case of MAICl and MAI − H ionic liquids, no charged species corresponding to the free *N*‐heterocyclic carbene was detected. On the contrary, in the presence of a sufficiently basic anion, such as acetate of MAIAc ionic liquid, an intense signal related to the free carbenic species was observed without the addition of an external base.

**Conclusions:**

*In situ* formation of free *N*‐heterocyclic carbenes from Brønsted acidic ionic liquids was demonstrated, highlighting the crucial role of anion basicity in promoting the C2‐H proton abstraction from imidazolium cations with a carboxylic side chain.

## 1 INTRODUCTION

In recent years, task‐specific Brønsted acidic ionic liquids (BAILs) have become increasingly popular and widely used in industrial processes since, as non‐volatile materials, they are considered less harmful and corrosive than traditional liquid acids.^1^, ^2^ The presence of carboxylic acid groups in the cation scaffold of BAILs represents indeed an important opportunity for the discovery of novel applications and new materials.^3^, ^4^, ^5^

Although BAILs are not liquid at room temperature and therefore cannot be used as reaction media,^6^ they are still of great importance in organocatalysis.^7^ Similarly to classic imidazolium‐based ionic liquids (ILs),^8^, ^9^ imidazolium BAILs can be a source of *N*‐heterocyclic carbenes (NHCs) generated from acidic imidazolium cations by a C2‐deprotonation reaction. Once formed, these species can act as basic or nucleophilic catalysts enabling innovative strategies to improve the effectiveness of chemical syntheses.^10^

Interestingly, in the presence of a sufficiently basic anion (e.g. acetate), the IL cation is supposed to form *in situ* catalytic amounts of NHC,^11^ as also demonstrated by the excellent yields of products in NHC‐catalysed reactions without the addition of an external deprotonating agent (e.g. strong bases).^12^, ^13^ Accordingly, the occurrence of endogenous free NHCs in room temperature ILs has long been experimentally and theoretically discussed.^14^ Also, an electrochemical study probed the crucial effect of the temperature in shifting the cation–NHC equilibrium towards the generation of free carbene.^15^, ^16^ However, to date, no spectroscopic evidence for the direct formation of NHCs in neat ILs has been reported.

Other than the above‐described chemical advantages of BAILs, the acidic carboxylic group inserted on the *N*‐side chains of the cation can act as a ‘charge tag’ for the direct detection of NHCs in ILs by mass spectrometry (MS), otherwise blind to intrinsically neutral species.^17^, ^18^, ^19^ In this context, the coupling of MS with soft ionization techniques, such as electrospray ionization (ESI), allows one to intercept elusive intermediates, and gently transfer them from solution to the gas‐phase environment for structural and reactivity investigations.^20^, ^21^, ^22^ Therefore, the chemistry of NHCs has been assessed in the gas phase,^23^, ^24^, ^25^, ^26^, ^27^ also in the view of knowing their detailed reaction mechanism to gain desirable benefits for solution chemistry. Free NHCs can be obtained in the gas phase, as well as in solution, by adding strong bases^28^, ^29^ or eventually forcing a hydrogen transfer reaction by collisionally dissociating the ion pair of the parent IL.^30^ Indirect evidence of the presence of NHCs in ILs was adduced by the addition of an aldehydic substrate to form the corresponding Breslow intermediate,^13^ by trapping transient NHCs in stable metal complexes,^31^, ^32^ or by stabilizing this species through a hydrogen bond between the electron pair of the carbene and the C2‐H hydrogen of a surrounding imidazolium cation.^33^ In the latter case, diazolium ILs were successfully employed in the gas phase as a source of incipient NHCs since the flexibility of the chemical linker connecting the two heterocyclic heads (one carbenic and the other cationic) allows the folding of the molecular structure and the formation of an intramolecular hydrogen bond.^34^, ^35^

**SCHEME 1:**
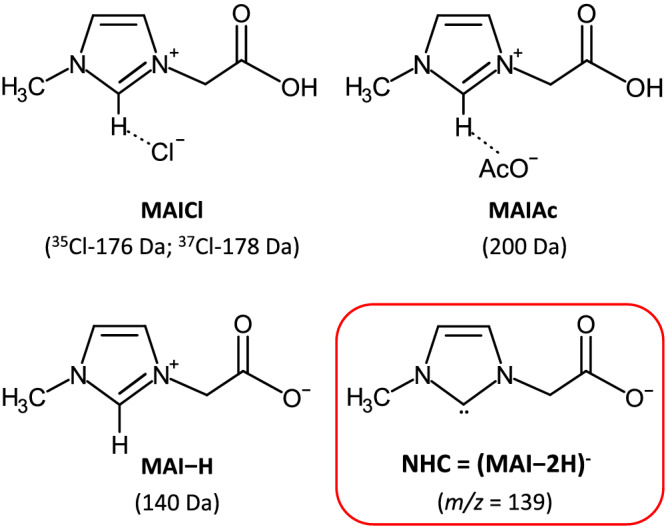
BAILs synthesized in this work and their molecular mass (Da). In the inset, the structure of the carbene obtained by C2‐H deprotonation and its *m/z* value are reported [Color figure can be viewed at wileyonlinelibrary.com]

We now report the straightforward detection of free NHCs in neat BAILs by mass spectrometric techniques. To this end, we have synthesized the following BAILs: 1‐methyl‐3‐carboxymethylimidazolium chloride (MAICl), 1‐methyl‐3‐carboxymethylimidazolium acetate (MAIAc) and the corresponding 2‐(3‐methyl‐1*H*‐imidazol‐3‐ium‐1‐yl)acetate zwitterion (MAI − H) (Scheme 1). Once interrogated by ESI‐MS in the negative ion mode, these compounds show a speciation that provides clear experimental evidence for the formation of NHCs, strongly influenced by the basicity of the anion.

## 2 MATERIALS AND METHODS

### 2.1 Chemicals and reagents

Starting compounds were commercially available (Sigma‐Aldrich) and used as received. All solvents (HPLC‐grade water, HPLC‐grade acetonitrile) were purchased from Carlo Erba Reagents S.r.l. and used without further purification.

### 2.2 Syntheses of ILs

#### 2.2.1 Procedure for synthesis of MAICl

MAICl was synthesized according to a literature procedure.^6^ MAICl: Yield 80%. White solid, m.p. 210°C. ^1^H NMR (400 MHz, CD_3_OD): *δ* (ppm) = 8.96 (s, 1 H), 7.61 (s, 1 H), 7.59 (s, 1 H), 4.97 (s, 2 H) 3.98 (s, 3 H). ^13^C NMR (101 MHz, CD_3_OD): *δ* (ppm) = 168.9, 137.7, 123.6, 122.9, 50.6, 35.1.

### 2.3 Procedure for synthesis of MAI − H

In a typical procedure, a mixture of MAICl (0.5 mmol) and triethylamine (0.55 mmol) in dichloromethane (1 mL) was stirred at room temperature for 24 h under nitrogen atmosphere. The solid was filtered and washed with the same solvent used for the synthesis (3 × 1 mL) to give the product.^6^

MAI − H: Yield 90%. White solid, m.p. 271°C (decomp). ^1^H NMR (400 MHz, D_2_O): *δ* (ppm) = 8.88 (s, 1 H), 7.55 (s, 1 H), 7.53 (s, 1 H), 4.87 (s, 2 H), 3.95 (s, 3 H). ^13^C NMR (101 MHz, D_2_O): *δ* (ppm) = 172.3, 136.8, 123.3, 123.1, 51.8, 35.6.

### 2.4 Procedure for synthesis of MAIAc

An ionic exchange chromatography column was used to obtain hydroxide ion form of salts. A short glass column for chromatography of 0.18 m in length and 0.01 m in diameter was loaded with resin Amberlite® IRA‐400 chloride (strongly basic gel‐type resin, quaternary ammonium functionality) from Sigma‐Aldrich. This column was packed using deionized distilled water and washed until free of chloride (checked with AgNO_3_). The column was then slowly treated with 20 mL of 1 M NaOH basic solution. The column was washed with deionized distilled water (3 × 20 mL) until the eluent showed neutral pH. A solution of MAICl (1 mmol) in 2 mL of water was then introduced in the column containing the hydroxide exchange resin and then the resin was washed with water until 50 mL of solution was collected. The solvent was evaporated under reduced pressure and the resulting white solid was kept overnight under high vacuum. The white solid salt thus obtained was subjected to ion exchange with acetic acid (1.0 equiv.) in a stirring solution of anhydrous methanol (2 mL) for 6 h at room temperature. After completion, methanol was removed under reduced pressure to afford colourless crystalline solid.

MAIAc: Yield 80%. Colourless crystalline solid, m.p. 199–207. ^1^H NMR (400 MHz, CD_3_OD): *δ* (ppm) = 8.88 (s, 1 H), 7.56 (s, 1 H), 7.53 (s, 1 H), 4.77 (s, 2 H), 3.96 (s, 3 H), 2.01 (s, 3 H). ^13^C NMR (101 MHz, CD_3_OD): *δ* (ppm) = 174.2, 169.9, 137.5, 123.5, 122.6, 51.9, 34.9, 19.7.

### 2.5 Procedure for synthesis of ethyl (*E*)‐2‐cyano‐3‐(4‐methoxyphenyl)acrylate*E*

A mixture of *p*‐anisaldehyde (0.5 mmol), ethyl cyanoacetate (0.5 mmol) and a 10% amount of MAI − H was stirred at room temperature in a 1.5 mL tube for 24 h under solvent‐free conditions. Upon completion of the reaction (monitored using thin‐layer chromatography), the reaction mixture solidified in the vial. Then the solidified mixture was washed with cold water (5 mL) to remove the catalyst and evaporated under reduced pressure to obtain the product.

Ethyl (*E*)‐2‐cyano‐3‐(4‐methoxyphenyl)acrylate^36^: Yield 60%. Yellow crystalline solid, m.p. 79–81°C. ^1^H NMR (200 MHz, CDCl_3_): *δ* (ppm) = 8.16 (s, 1 H), 7.99 (d, *J* = 9.0 Hz, 2 H), 6.98 (d, *J* = 8.8 Hz, 2 H), 4.36 (q, *J* = 7.0 Hz, 2 H), 3.89 (s, 3 H), 1.38 (t, *J* = 7.0 Hz, 3 H). ^13^C NMR (50.3 MHz, CDCl_3_): *δ* (ppm) = 163.7, 162.9, 154.2, 133.5, 124.2, 116.1, 114.7, 99.2, 62.3, 55.6, 14.1.

All the products except MAIAc are known compounds and were identified by comparison of their spectroscopic data with those reported. The new compound was properly characterized by its spectroscopic data: ^1^H NMR and ^13^C NMR spectra (Figures S1–S4, supporting information).

NMR spectra were recorded at ambient temperature with a Bruker Avance spectrometer (400 MHz) or a Spinsolve 60 spectrometer operating at 60 MHz, using the solvent as internal standard. The chemical shifts (*δ*) are given in ppm relative to tetramethylsilane. An SMP2 (Stuart Science) apparatus was employed to measure the melting points of the synthesized compounds.

### 2.6 MS experiments

MS experiments were performed using an AmaZon SL ion trap (Bruker Daltonics, Bremen, Germany) equipped with ESI and atmospheric pressure chemical ionization (APCI) sources operating in the positive or negative ion mode. Typical experimental conditions were as follows: capillary, ±4 kV; endplate offset, ±400 V; nebulizer (N_2_), 5.0 psi; dry gas (N_2_), 2.5 L min^−1^; dry temperature, 200°C.

Other instrumental parameters, such as RF level, trap drive and the discharge current for the APCI process, were in turn optimized to ensure maximum ion transmission in the *m*/*z* range of interest. The acidic ILs MAICl, MAI − H and MAIAc were dissolved in a mixture of H_2_O and CH_3_CN (1:5, V/V) at millimolar concentration and infused into the ESI or APCI source by the onboard syringe pump at a flow rate ranging between 5 and 20 μL min^−1^ depending on the source mounted on the instrument. Full‐scan mass spectra were acquired in the 50–800 *m*/*z* range as an average of 50 scans using Compass DataAnalysis software supplied with the instrument.

Low‐energy collision‐induced dissociation (CID) was performed by applying an excitation AC voltage to the end caps of the trap to induce multiple low‐energy collisions of the trapped ions with helium buffer gas. The resonance excitation voltage was applied for 30 ms at an amplitude (indicated in the caption of selected CID spectra) that allowed a reduction of the parent ion intensity to 30–50%, a fragmentation delay of 10 ms and a standard cut‐off of 27%. The ionic species of interest were isolated with a width of 1 *m*/*z* or greater to stabilize large‐size cluster ions. The same parameters were set for any further step of isolation and fragmentation (MS^*n*^) inserted into the scan sequence to assess the complete dissociation pattern of the ionic species under investigation.

## 3 RESULTS AND DISCUSSSION

The gas‐phase behaviour of the task‐specific MAICl IL has been investigated in the positive ion mode using ESI‐MS techniques by Mota et al,^37^ revealing aggregation phenomena characteristic of saline compounds^38^, ^39^ and analogous imidazolium‐based ILs.^30^ Similar ESI‐(+) mass spectra were also obtained in our case and reported in Figure S5 (supporting information) since not being indicative of the presence of NHCs in the neat ILs under study.

On the contrary, it is of greatest interest to investigate the speciation of these salts in negative polarity, although commonly less explored than the positive one.^40^, ^41^, ^42^

**FIGURE 1:**
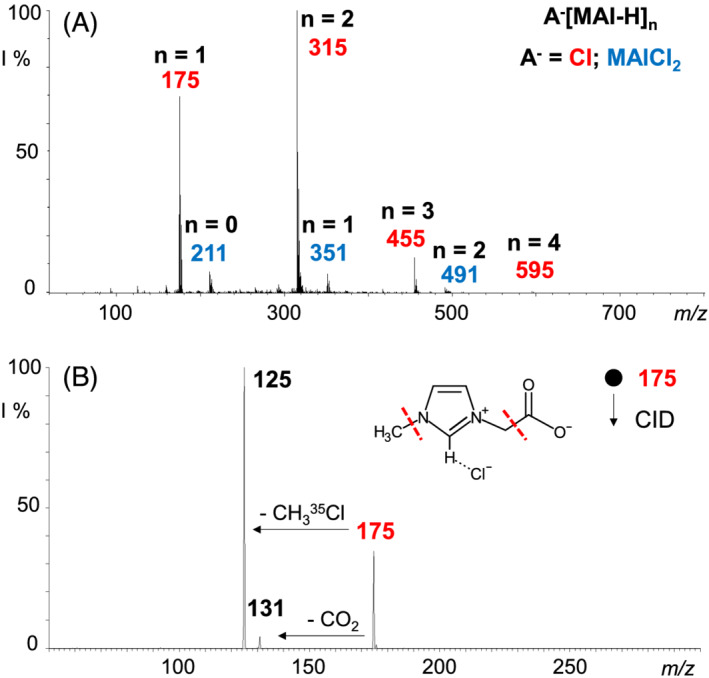
(A) ESI‐(−) mass spectrum of the MAICl IL and (B) ESI‐(−) CID mass spectrum of the ion at *m/z* 175, corresponding to ^35^Cl^−^[MAI − H]. Fragmentation amplitude 0.27 V [Color figure can be viewed at wileyonlinelibrary.com]

Accordingly, the ESI‐(−) mass spectrum of MAICl in the 50–800 *m*/*z* range is characterized by ionic aggregates of the general formula A^−^[MAI − H]_*n*_ with *n* = 1–4, showing chloride ion as a clustering anion A (Figure 1A). Ionic species of the type MAICl_2_^−^[MAI − H]_*n*_ (*n* = 0–2) result in a minor series of 36 Da right‐shifted with respect to the corresponding Cl^−^[MAI − H]_*n* + 1_ ionic peaks. Mass‐*to*‐charge attribution was verified by the characteristic ^35^Cl/^37^Cl isotope pattern and CID experiments, describing these species as a series of [MAI − H] zwitterions coordinated to a Cl^−^ core (Figure S6, supporting information). It is worth noting that no ion at *m*/*z* 139, corresponding to the negatively charged NHC [MAI − 2H]^−^, was detected in the MAICl mass spectrum. The chloride anion, in fact, is not able to deprotonate the C2 carbon of the MAI − H zwitterion neither in solution nor in the gas phase, as also demonstrated by the CID mass spectrum of the ionic species at *m*/*z* 175 corresponding to a charged cluster between the neutral MAI − H zwitterion and the chloride anion, Cl^−^[MAI − H], displayed in Figure 1B. The cleavage of the imidazolium side chains, exemplified by the alternative loss of a CO_2_ (44 Da, fragment ion at *m*/*z* 131) or a CH_3_^35^Cl (50 Da, fragment ion at *m*/*z* 125) portion, prevails over the release of the H^35^Cl (36 Da) neutral counterpart since no [MAI − 2H]^−^ ion at *m*/*z* 139 was observed, even providing high collision energies. The same fragmentation channels were also probed for the ^37^Cl‐isotopologue, thus confirming the attribution of both product ions and the actual lack of the diagnostic ion at *m*/*z* 139 (Figure S7, supporting information).

Since the p*K*_a_ value for the deprotonation of C2‐H is in the 21–23 range,^43^ the only way to obtain a significant amount of carbene from MAICl is by adding a strong base to the IL solution,^29^, ^44^ such as potassium *tert*‐butoxide (KOtBu; p*K*_a_ = 17)^45^ or 1,8‐diazabicyclo[5.4.0]undecane (DBU; p*K*_a_ = 13.5).^46^ The latter is also considered a ‘super base’ in the gas phase, since its proton affinity (PA) exceeds 239 kcal mol^−1^ (DBU, PA = 250.45 kcal mol^−1^), thus allowing the measurement of important gas‐phase thermochemical properties of NHCs.^47^

**FIGURE 2:**
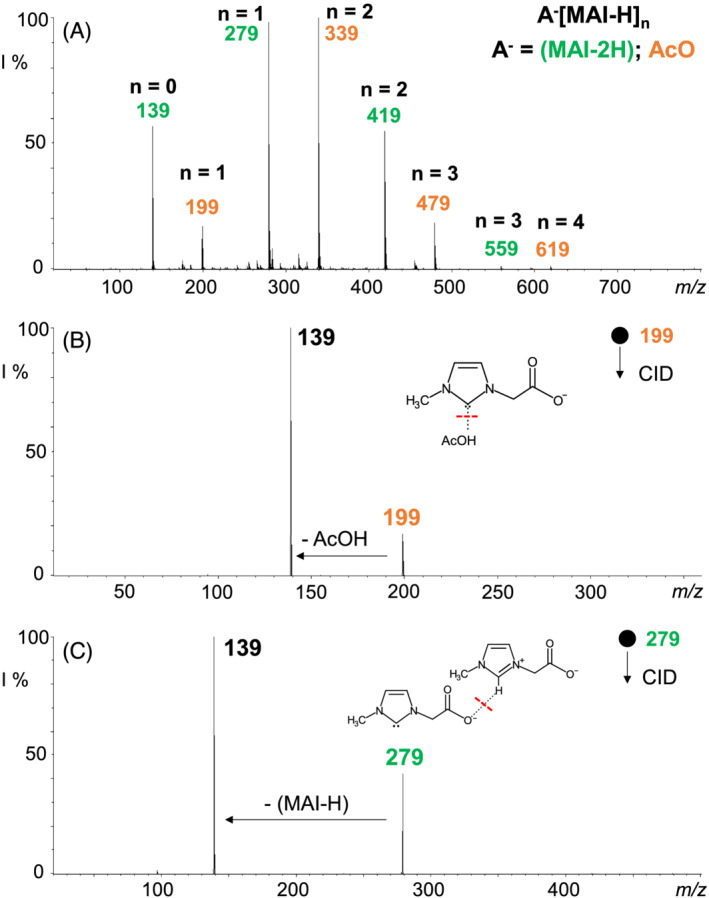
(A) ESI‐(−) mass spectrum of the MAIAc IL, (B) ESI‐(−) CID mass spectrum of the ion at *m/z* 199, fragmentation amplitude 0.12 V, and (C) ESI‐(−) CID mass spectrum of the ion at *m/z* 279, fragmentation amplitude 0.35 V [Color figure can be viewed at wileyonlinelibrary.com]

Interestingly, passing to the ESI‐(−) mass spectrometric analysis of MAIAc IL under soft ionization conditions (Figure 2A), an intense ion at *m*/*z* 139, [MAI − 2H]^−^, attributable to the free negatively charged carbene, was detected. To the best of our knowledge, this is the first time that a free carbene has been observed from a BAIL, such as MAIAc. The carbenic species [MAI − 2H]^−^ can reasonably originate from C2‐H deprotonation of the MAI − H zwitterion performed in solution by the acetate anion (p*K*_a_ AcOH = 4.76).^48^, ^49^

Moreover, the fragmentation pattern of the ion at *m*/*z* 139 is consistent with that of [MAI − 2H]^−^ obtained by the DBU‐deprotonation of MAI cation in MAICl (Figure S8, supporting information).

The MAIAc spectrum of Figure 2A also shows two series of ionic aggregates of the general formula A^−^[MAI − H]_*n*_ (A^−^ = [MAI − 2H]^−^ with *n* = 1–3; A^−^ = AcO^−^ with *n* = 1–4), in which the MAI − H zwitterion is clustered by either AcO^−^ or [MAI − 2H]^−^. A close inspection of the CID mass spectrum of the AcO^−^[MAI − H] cluster ion at *m*/*z* 199 (Figure 2B) shows only a fragmentation channel consisting of the easy release of an AcOH (60 Da) portion giving rise to the negatively charged NHC [MAI − 2H]^−^ at *m*/*z* 139. Fragment ions originating from the breakage of the imidazole scaffold were indeed not observed, contrary to the corresponding ionic aggregate Cl^−^[MAI − H] at *m*/*z* 175 (Figure 1B). Therefore, the ionic species at *m*/*z* 199 can be described as an acetic acid–carbene aggregate of the AcOH·[MAI − 2H]^−^ type. Likewise, the CID spectra from larger clusters (*m*/*z* 339 and 479) show preferential loss of acetic acid over that of zwitterion (Figure S9, supporting information). The ‘naked’ [MAI − 2H]^−^ ion in the ESI‐(−) full‐scan mass spectrum of MAIAc IL could in principle result from an in‐source fragmentation process of the AcOH·[MAI − 2H]^−^ precursor species, also considering the low excitation voltage necessary to dissociate the cluster ion at *m*/*z* 199 (see Figure 2B). The source voltages may indeed act on AcOH·[MAI − 2H]^−^ ionic aggregate by further shifting the acid–base equilibrium towards the formation of [MAI − 2H]^−^. Nevertheless, in the ESI‐(−) mass spectrum of MAIAc, the intense ionic distribution of ([MAI − 2H]·[MAI − H]_*n*_)^−^ (*n* = 0–3) cluster ions accounts for the formation of free carbene in solution and its solvation by *n* units of MAI − H zwitterions. Considering the first cluster of the series, the ([MAI − 2H]·[MAI − H])^−^ ion at *m*/*z* 279, it is undoubtedly composed of a [MAI − 2H]^−^ carbene and a zwitterionic unit, as demonstrated by its CID mass spectrum reported in Figure 2C (see also Figure S9, supporting information).

A further question is whether the deprotonated carboxylic lateral chain of the MAI − H zwitterion can generate the corresponding carbene. However, the p*K*_a_ value for the acidic lateral chain of the MAI cation of 1.90, about 2.5 times lower than that of free acetate,^6^ would exclude this possibility, in agreement with the experimental results. Predictably, the mass spectrum of the MAI − H solution in the negative ion mode did not show any ionic signal owing to the neutral nature of MAI − H species, not even the ion at *m*/*z* 139, thus excluding the occurrence of a proton transfer reaction between two zwitterion moieties.

Finally, to obtain more information about the chemical properties of neutral MAI − H zwitterion, we used this compound as a catalyst in the Knoevenagel condensation. In this regard, we have recently highlighted two different mechanisms for this reaction depending on the chemical features of the IL.^50^ In the presence of chloride‐based ILs, such as MAICl, the condensation proceeds through a classic base‐catalysed pathway characterized by the formation of an aldolic intermediate between the aldehyde and the activated methylene substrate. On the contrary, in the presence of acetate‐based ILs, such as 1‐butyl‐3‐methylimidazolium acetate, the incipient carbene drives the reaction to the final product by adding the aldehydic carbonyl and giving rise to the Breslow intermediate.

In this case, by reacting *p*‐anisaldehyde, ethyl cyanoacetate and 10% mol of MAI − H zwitterion catalyst under the same experimental conditions previously used,^36^ we only detected the negatively charged aldolic intermediate characteristic of the classic base‐catalysed mechanism (Figure S10, supporting information). No Breslow intermediate between the [MAI − 2H]^−^ carbene and the aromatic aldehyde was intercepted, thus excluding even the possible shift of the equilibrium towards the NHC in the presence of *p*‐anisaldehyde. The evolution of the aldolic intermediate into the corresponding ethyl (*E*)‐2‐cyano‐3‐(4‐methoxyphenyl)acrylate was verified by its isolation in 60% yield and characterization by ^1^H NMR, ^13^C NMR and MS (Figures S4 and S11, supporting information). Other synthetic applications with the MAIAc catalyst synthesised in the work reported in this paper are currently underway.

## 4 CONCLUSIONS

We have reported herein the direct evidence of a free NHC in a BAIL. Considering the deep‐rooted use of NHCs in synthetic chemistry and catalysis, the question related to the presence of these reactive species in the reaction medium is still of utmost importance.

This experimental study demonstrates the possibility of using acetate anions to furnish proper amounts of stable NHCs from BAILs, possibly also with different acetate‐based ILs opportunely designed for task‐specific applications. The ESI‐(−) mass spectrometric analysis of MAIAc IL allowed us to detect the presence of free NHC, pointing out that its generation in MAIX is strongly affected by the basicity of the anion (X = AcO or Cl). This result paves the way for the use of the novel MAIAc and other customized BAILs as catalysts in carbene‐mediated reactions, avoiding the use of other bases with important benefits for organic synthesis, and highlights the usefulness of MS studies in the detection of highly reactive species.

## Supporting information


**Figure S1.** ^1^H‐NMR and ^13^C‐NMR spectra of MAICl ionic liquid.
**Figure S2.** ^1^H‐NMR and ^13^C‐NMR spectra of MAI‐H ionic liquid.
**Figure S3.** ^1^H‐NMR and ^13^C‐NMR spectra of MAIAc ionic liquid.
**Figure S4.** ^1^H‐NMR and ^13^C‐NMR spectra of Ethyl (*E*)‐2‐cyano‐3‐(4‐methoxyphenyl) acrylate.
**Figure S5.** ESI‐(+) mass spectra of a) MAICl, b) MAI‐H zwitterion, and c) MAIAc ionic liquids.
**Figure S6.** ESI‐(−) CID mass spectra of the ions a) at *m/z* 315 (frag. Ampl. 0.15 V) and b) at *m/z* 455 (frag. Ampl. 0.19 V) corresponding to Cl^‐^[MAI‐H]_2_ and Cl^‐^[MAI‐H]_3_ clusters, respectively.
**Figure S7.** ESI‐(−) CID mass spectrum of the ion at *m/z* 177 corresponding to ^37^Cl‐[MAI‐H] species. Fragmentation amplitude 0.27 V.
**Figure S8.** ESI‐(−) CID mass spectrum of the ion at *m/z* 139 isolated from a solution of a) MAICl + DBU (1:1) and b) MAIAc dissolved in H_2_O/CH_3_CN. Fragmentation amplitude in both spectra 0.50 V.
**Figure S9.** ESI‐(−) CID mass spectra of the ions a) at *m/z* 339 (frag. Ampl. 0.10 V), b) at *m/z* 419 (frag. Ampl. 0.12 V), and c) at *m/z* 479 (frag. Ampl. 0.12 V) corresponding to AcOH·(MAI‐2H)^‐^[MAI‐H], (MAI‐2H)^‐^[MAI‐H]_2_, and AcOH·(MAI‐2H)^‐^[MAI‐H]_2_ clusters, respectively.
**Figure S10.** ESI‐(−) mass spectrum of a 1:1 *p*‐anisaldehyde and ethyl cyanoacetate reaction mixture in the presence of 10% amount of MAI‐H catalyst.
**Figure S11.** APCI‐(−) CID mass spectra of a) the Knoevenagel product at *m/z* 231 and b) its fragment ion at *m/z* 216.

## Data Availability

The data that supports the findings of this study are available in the supplementary material of this article.
