# Supplementary material for: Free N‐heterocyclic carbenes from Brønsted acidic ionic liquids: Direct detection by electrospray ionization mass spectrometry
Source: Rapid Commun Mass Spectrom. 2022 Jul 7;36(17):e9338. doi: 10.1002/rcm.9338 (PMC9542177; doi:10.1002/rcm.9338)
Supplement: Supplementary file 1 — Figure S1. 1H‐NMR and 13C‐NMR spectra of MAICl ionic liquid. Figure S2. 1H‐NMR and 13C‐NMR spectra of MAI‐H ionic liquid. Figure S3. 1H‐NMR and 13C‐NMR spectra of MAIAc ionic liquid. Figure S4. 1H‐NMR and 13C‐NMR spectra of Ethyl (E)‐2‐cyano‐3‐(4‐methoxyphenyl) acrylate. Figure S5. ESI‐(+) mass spectra of a) MAICl, b) MAI‐H zwitterion, and c) MAIAc ionic liquids. Figure S6. ESI‐(−) CID mass spectra of the ions a) at m/z 315 (frag. Ampl. 0.15 V) and b) at m/z 455 (frag. Ampl. 0.19 V) corresponding to Cl‐[MAI‐H]2 and Cl‐[MAI‐H]3 clusters, respectively. Figure S7. ESI‐(−) CID mass spectrum of the ion at m/z 177 corresponding to 37Cl‐[MAI‐H] species. Fragmentation amplitude 0.27 V. Figure S8. ESI‐(−) CID mass spectrum of the ion at m/z 139 isolated from a solution of a) MAICl + DBU (1:1) and b) MAIAc dissolved in H2O/CH3CN. Fragmentation amplitude in both spectra 0.50 V. Figure S9. ESI‐(−) CID mass spectra of the ions a) at m/z 339 (frag. Ampl. 0.10 V), b) at m/z 419 (frag. Ampl. 0.12 V), and c) at m/z 479 (frag. Ampl. 0.12 V) corresponding to AcOH·(MAI‐2H)‐[MAI‐H], (MAI‐2H)‐[MAI‐H]2, and AcOH·(MAI‐2H)‐[MAI‐H]2 clusters, respectively. Figure S10. ESI‐(−) mass spectrum of a 1:1 p‐anisaldehyde and ethyl cyanoacetate reaction mixture in the presence of 10% amount of MAI‐H catalyst. Figure S11. APCI‐(−) CID mass spectra of a) the Knoevenagel product at m/z 231 and b) its fragment ion at m/z 216. [file RCM-36-e9338-s001.pdf]

Supporting Information

Free *N*-heterocyclic carbenes from Brønsted acidic ionic liquids: direct detection by electrospray ionization mass spectrometry

Chiara Salvitti,*[1] Federico Pepi,[1] Marta Managò,[1] Martina Bortolami,[2] Cinzia Michenzi,[2] Isabella Chiarotto,*[2] Anna Troiani,*[1] Giulia de Petris[1]

1. Dipartimento di Chimica e Tecnologie del Farmaco, Sapienza Università di Roma, P.le Aldo Moro 5, 00185 Roma, Italy. E-mail: anna.troiani@uniroma1.it; chiara.salvitti@uniroma1.it

2. Dipartimento di Scienze di Base e Applicate per l'Ingegneria, Sapienza Università di Roma, Via Castro Laurenziano 7, 00161 Roma, Italy. E-mail: isabella.chiarotto@uniroma1.it

Table of Content:

^{1}H-NMR and ^{13}C-NMR spectra (Figures S1-S4) pg. 2

Mass spectra (Figures S5-S11) pg. 6

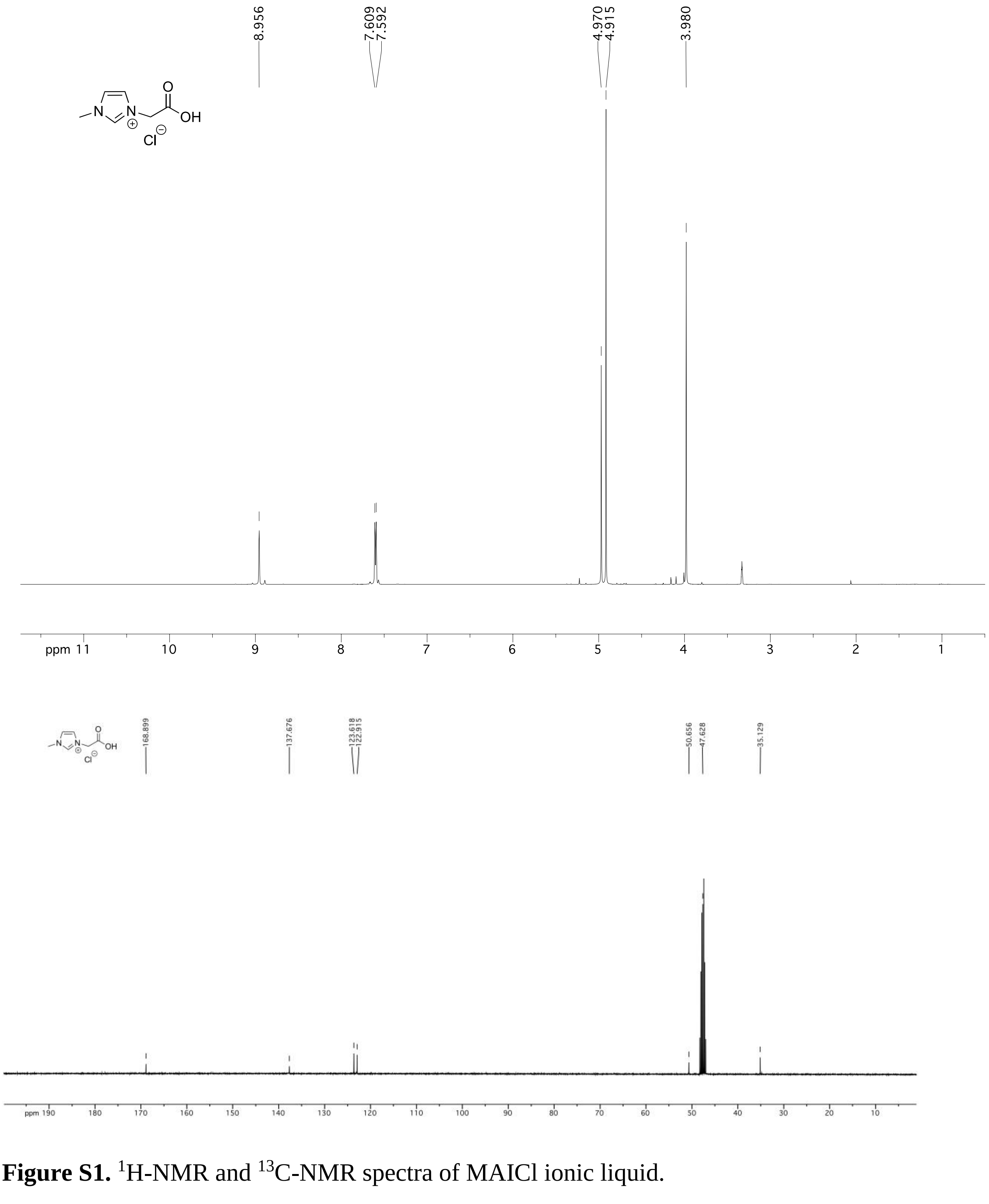

Figure S1. ^{1}H-NMR and ^{13}C-NMR spectra of MAICl ionic liquid.

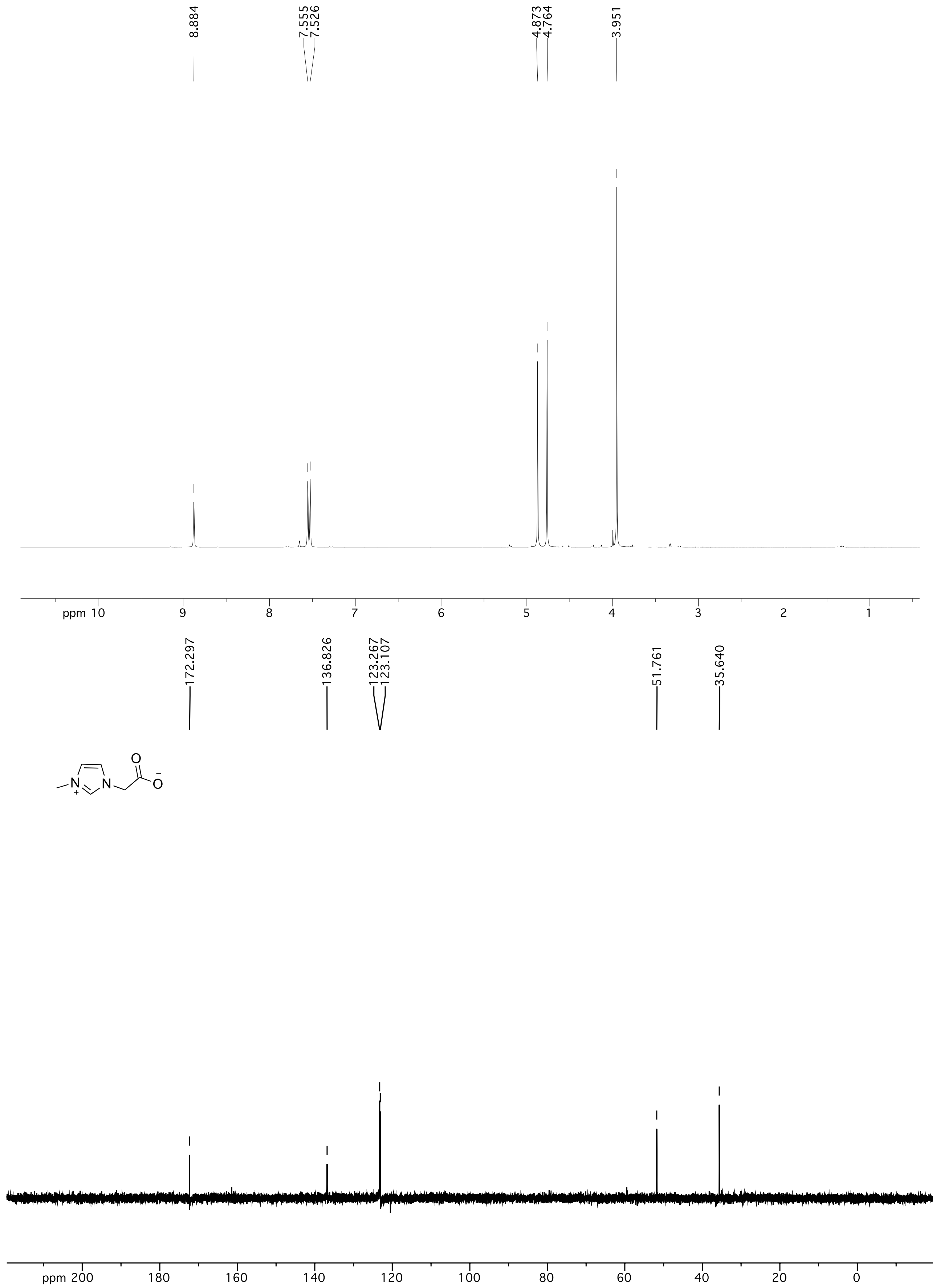

Figure S2. ^{1}H-NMR and ^{13}C-NMR spectra of MAI-H ionic liquid.

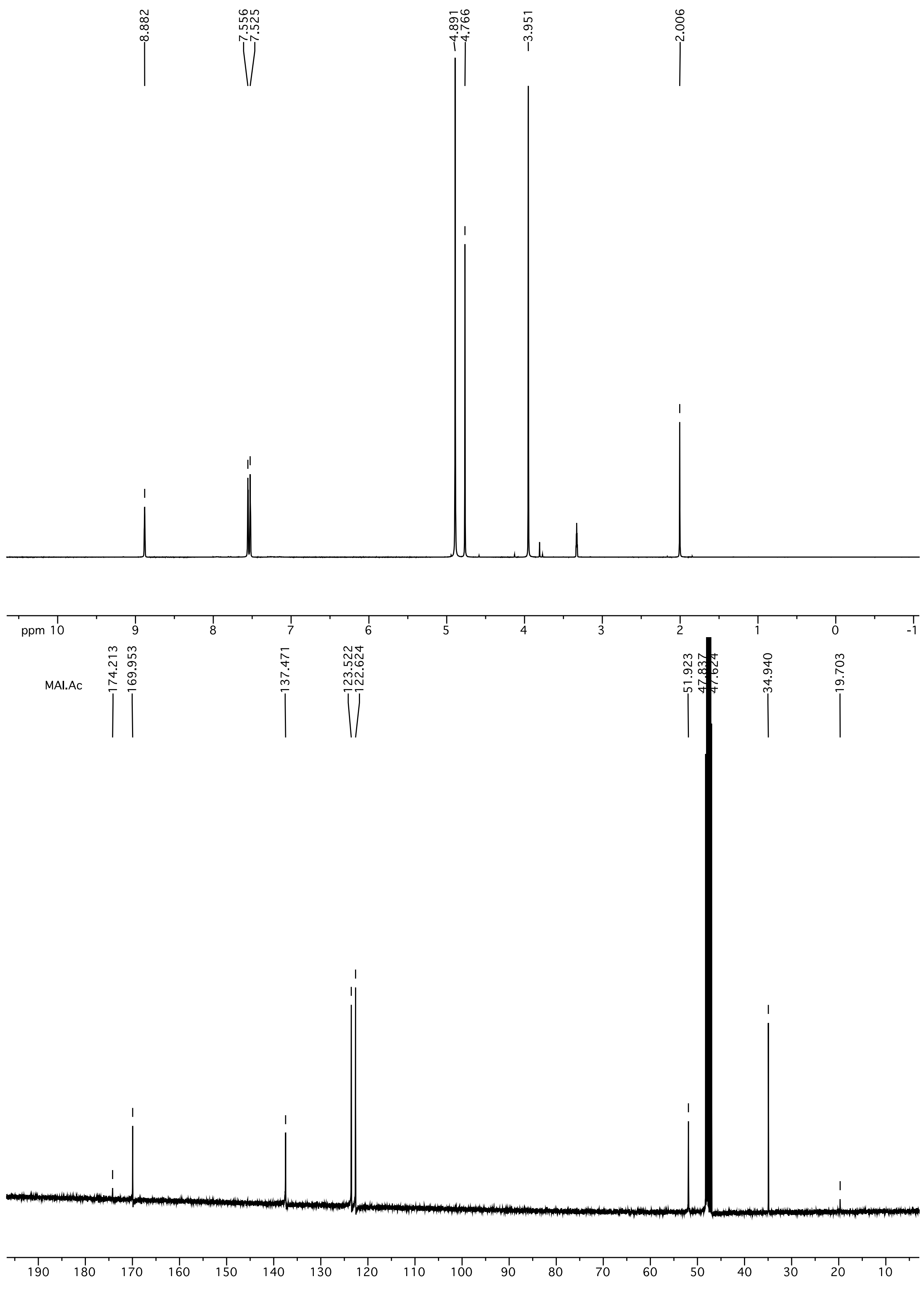

Figure S3. ^{1}H-NMR and ^{13}C-NMR spectra of MAIAc ionic liquid.

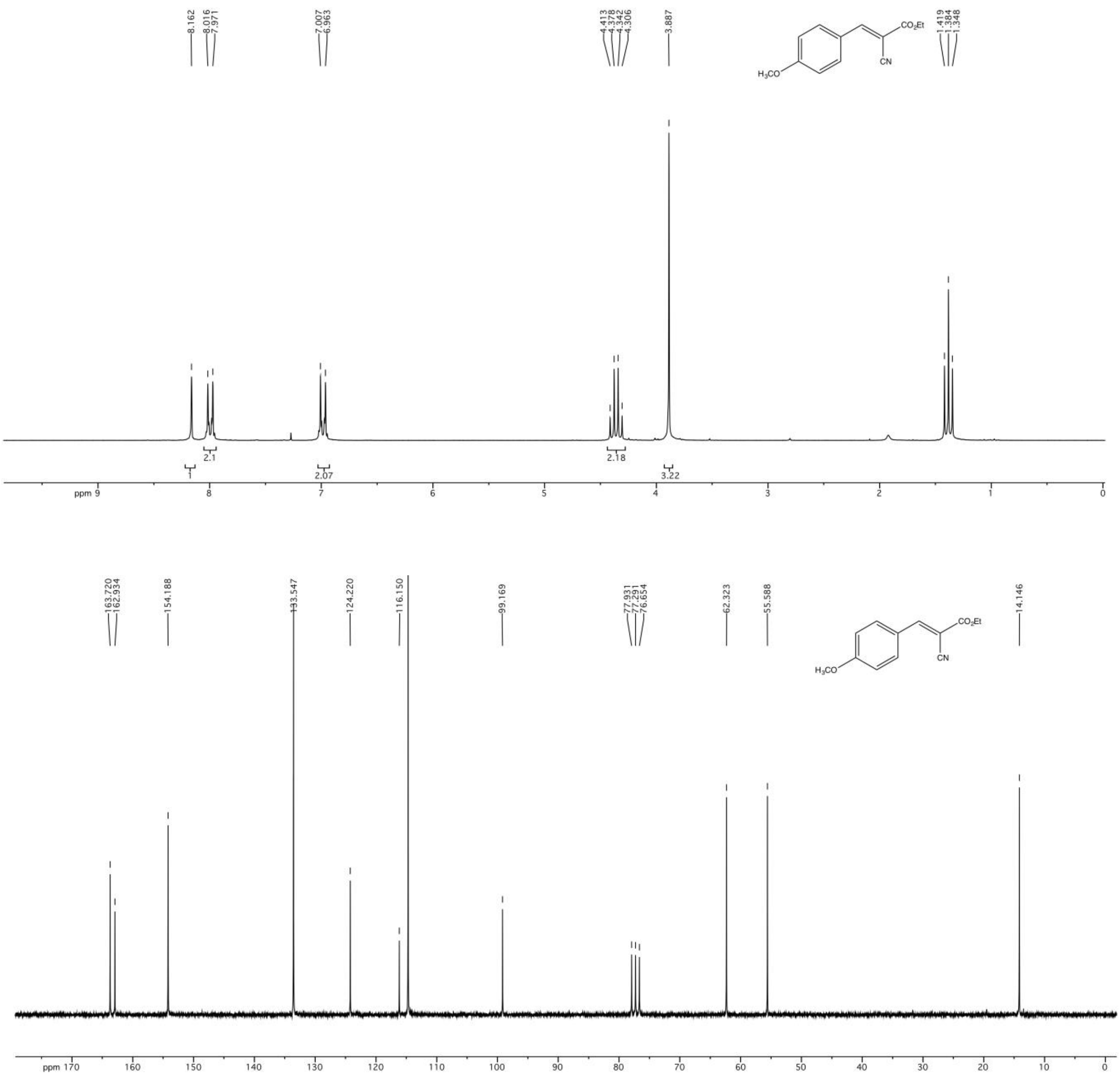

Figure S4. ^{1}H-NMR and ^{13}C-NMR spectra of Ethyl (*E*)-2-cyano-3-(4-methoxyphenyl) acrylate.

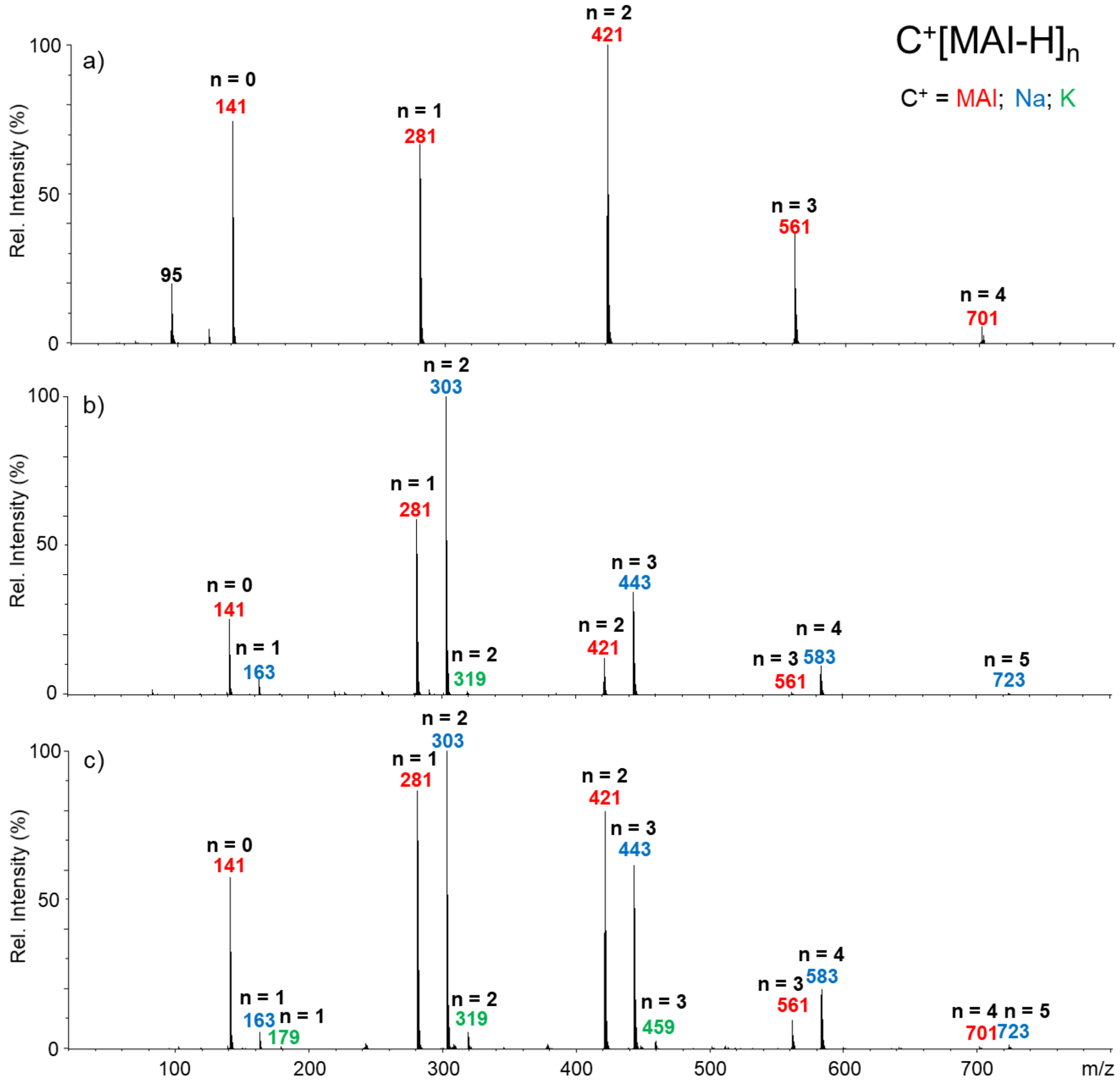

Figure S5. ESI-(+) mass spectra of a) MAICl, b) MAI-H zwitterion, and c) MAIAc ionic liquids.

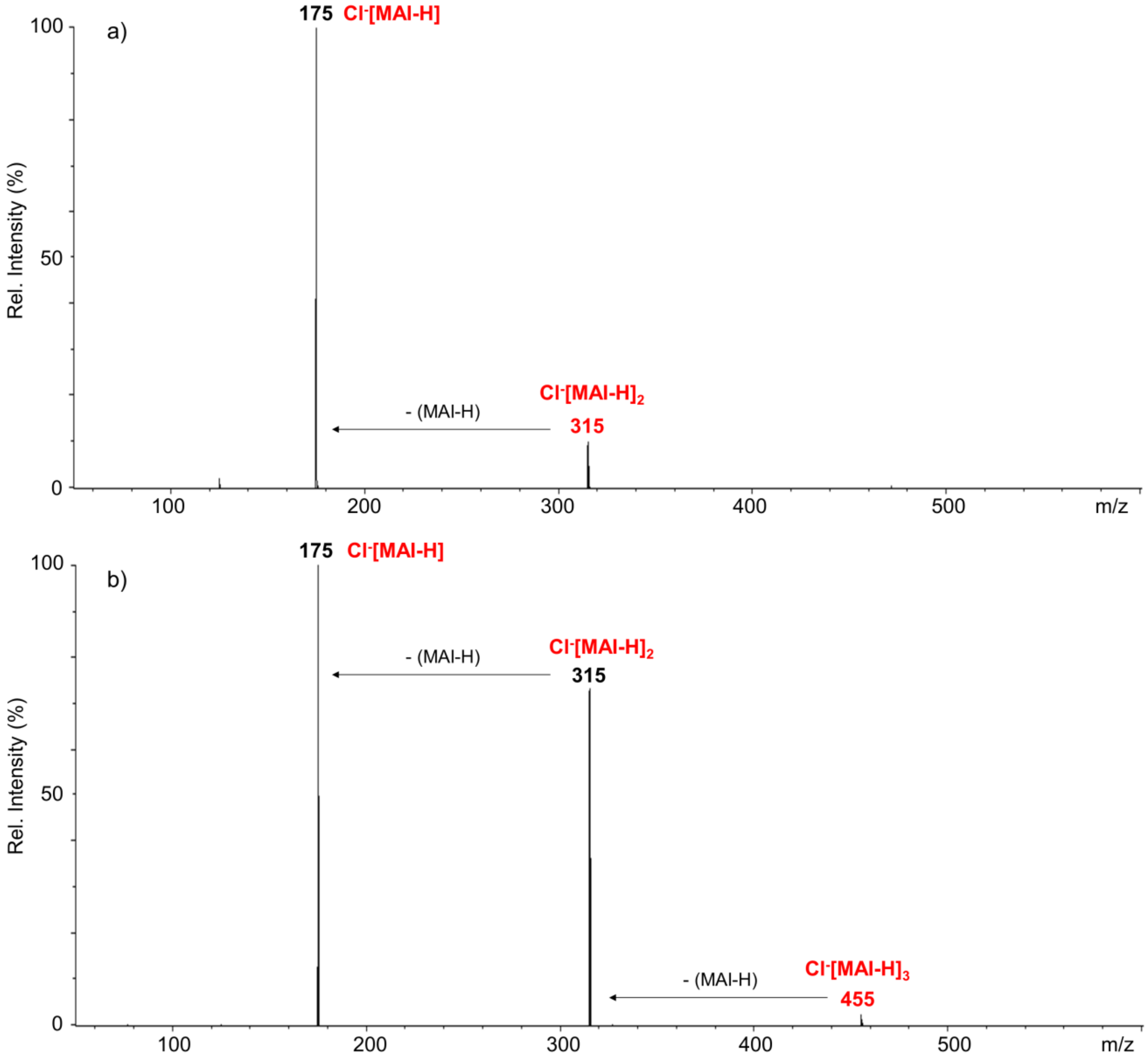

Figure S6. ESI-(-) CID mass spectra of the ions a) at *m/z* 315 (frag. ampl. 0.15 V) and b) at *m/z* 455 (frag. ampl. 0.19 V) corresponding to $Cl^-[MAI\text{-}H]_2$ and $Cl^-[MAI\text{-}H]_3$ clusters, respectively.

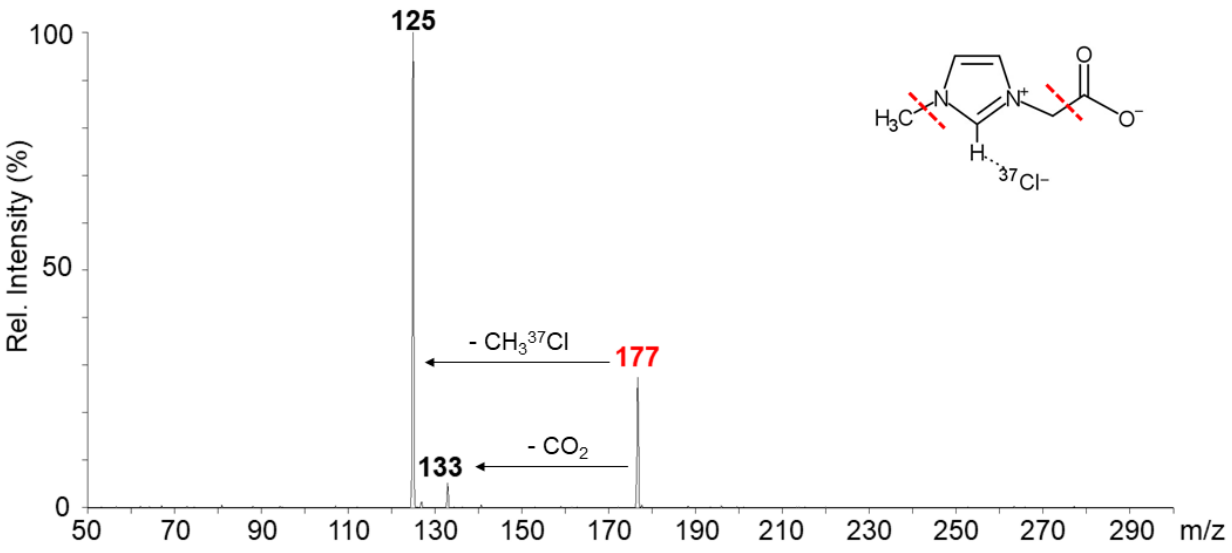

Figure S7. ESI-(-) CID mass spectrum of the ion at *m/z* 177 corresponding to $^{37}Cl^{-}$[MAI-H] species. Fragmentation amplitude 0.27 V.

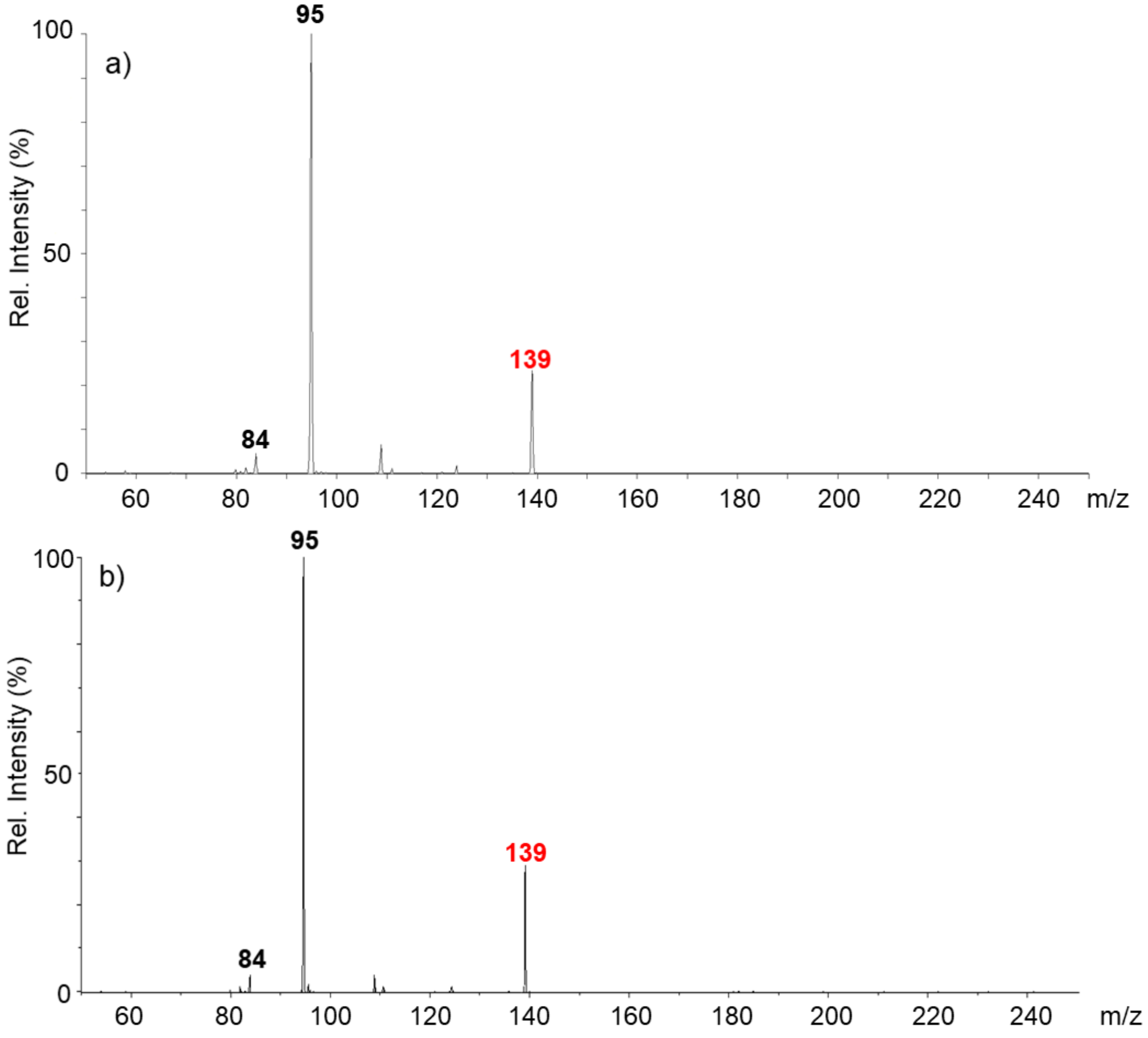

Figure S8. ESI-(-) CID mass spectrum of the ion at *m/z* 139 isolated from a solution of a) MAICl + DBU (1:1) and b) MAIAc dissolved in H_2O/CH_3CN. Fragmentation amplitude in both spectra 0.50 V.

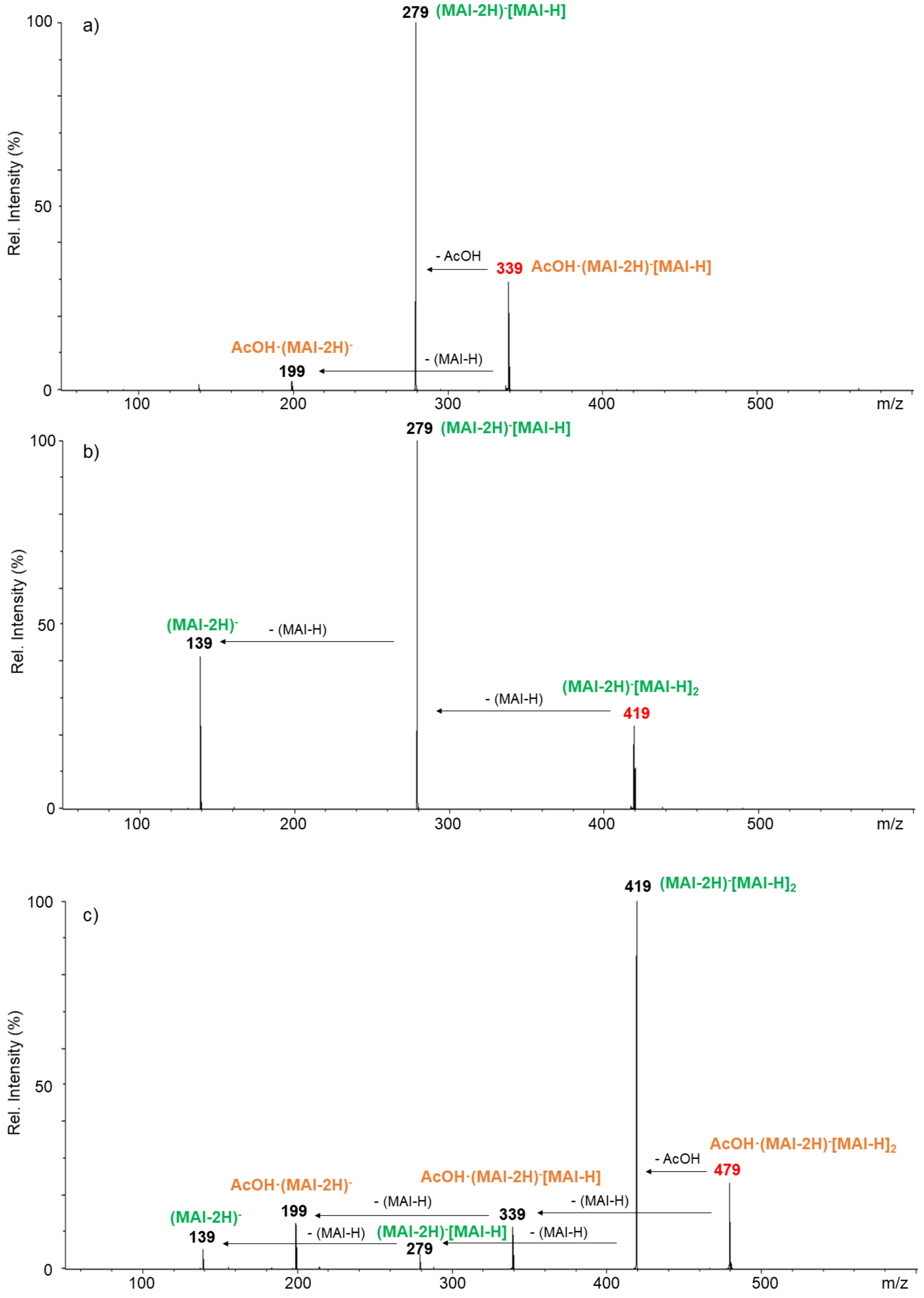

Figure S9. ESI-(-) CID mass spectra of the ions a) at *m/z* 339 (frag. ampl. 0.10 V), b) at *m/z* 419 (frag. ampl. 0.12 V), and c) at *m/z* 479 (frag. ampl. 0.12 V) corresponding to AcOH·(MAI-2H)$^-$[MAI-H], (MAI-2H)$^-$[MAI-H]$_2$, and AcOH·(MAI-2H)$^-$[MAI-H]$_2$ clusters, respectively.

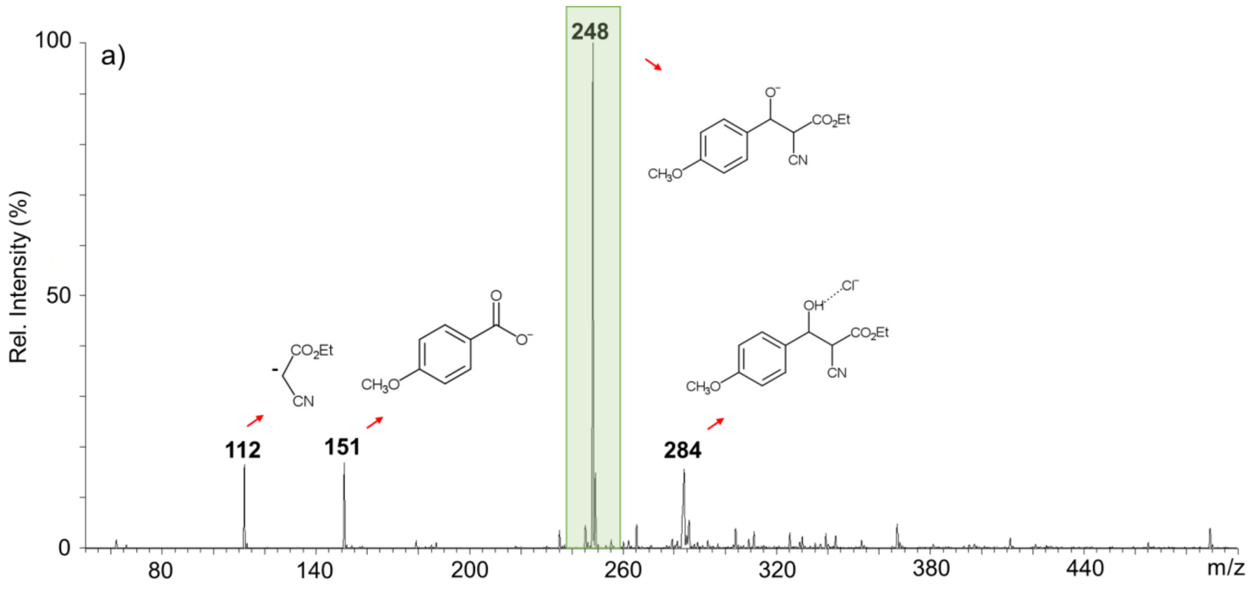

Figure S10. ESI-(-) mass spectrum of a 1:1 *p*-anisaldehyde and ethyl cyanoacetate reaction mixture in the presence of 10% amount of MAI-H catalyst.

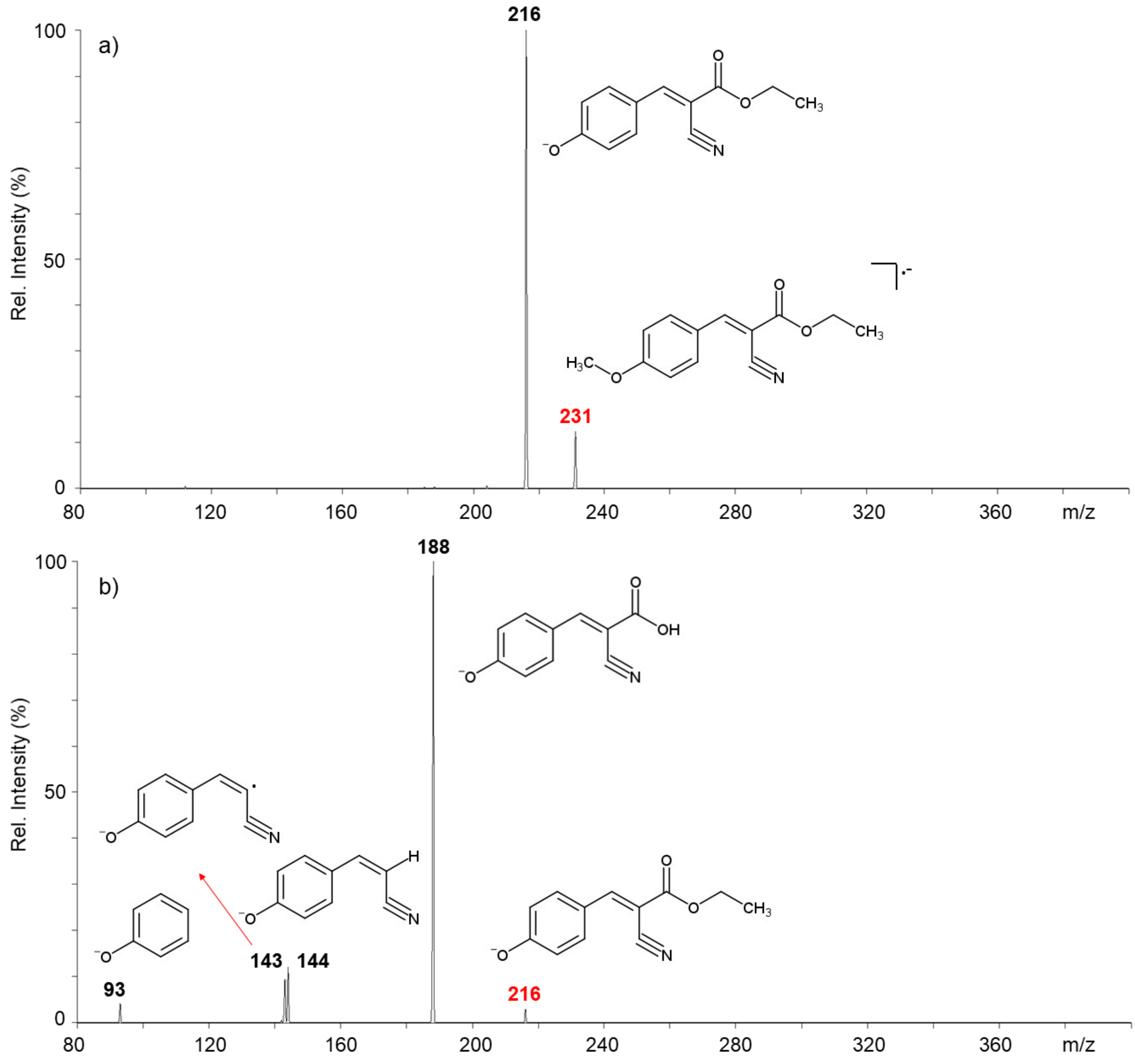

Figure S11. APCI-(-) CID mass spectra of a) the Knoevenagel product at *m/z* 231 and b) its fragment ion at *m/z* 216.
